# Evaluating Golden and Recurring Esthetic Dental (RED) Proportions Using Two Methods in an Urban Tirupati Population

**DOI:** 10.7759/cureus.113953

**Published:** 2026-08-04

**Authors:** Raja Reddy N, Krishnakanth Bagati, Tilak Vardhan Reddy K, Archana B, Sruthi Reddy C, Joe Prem Nishal C, Varsha Reddy D

**Affiliations:** 1 Department of Prosthodontics, CKS Theja Institute of Dental Sciences and Research, Tirupati, IND; 2 Department of Prosthodontics, Sibar Institute of Dental Sciences, Guntur, IND

**Keywords:** dental esthetics, maxillary anterior teeth, photographic analysis, prosthodontics, smile design

## Abstract

Background: The Golden Proportion and Recurring Esthetic Dental (RED) Proportion have been historically proposed as mathematical guidelines for achieving optimal smile esthetics. However, their applicability in natural dentition remains controversial because anterior tooth proportions vary among different populations. This study aimed to evaluate the presence of the Golden Proportion and RED Proportion in the Tirupati urban population and compared measurements obtained using photographic and cast-based methods.

Methods: This analytical cross-sectional study included 200 participants aged 18-30 years with natural maxillary anterior dentition. Mesiodistal widths of the maxillary central incisors, lateral incisors, and canines were measured using standardized digital photographic analysis and direct measurements on dental casts with a digital Vernier caliper. Golden Proportion and RED Proportion ratios were calculated and compared between the two methods. Continuous variables were expressed as mean ± standard deviation, and paired *t*-tests were used for comparisons. Statistical significance was set at *p* < 0.05.

Results: Central incisor measurements did not differ significantly between the photographic and cast-based measurement methods (*p* > 0.05), whereas significantly greater widths were recorded for the lateral incisors and canines using Vernier calipers (*p* < 0.001). Central and lateral incisors exhibited bilateral symmetry, while the canines showed minor asymmetry. The photographic method yielded a lateral incisor-to-central incisor ratio closer to the theoretical Golden Proportion; however, neither the Golden Proportion nor the RED Proportion was consistently identified in the study population.

Conclusions: Neither the Golden Proportion nor the RED Proportion was consistently observed in the maxillary anterior dentition of the Tirupati urban population, indicating that these concepts should not be considered universal standards for smile esthetics. Photographic and direct measurements provide complementary information by representing perceived and actual tooth dimensions, respectively. Individualized smile design based on patient-specific anatomical characteristics and clinical judgment may be more appropriate than strict adherence to mathematical proportional theories.

## Introduction

Smile esthetics is an important component of facial appearance and has a considerable influence on social interactions, self-confidence, and perceived attractiveness. Increasing awareness of cosmetic dentistry and the widespread use of digital media have further heightened patient expectations regarding an esthetic smile, making smile analysis an integral part of contemporary dental practice [1]. The maxillary anterior teeth play a central role in smile esthetics because they are the most visible during speech and smiling. Their size, shape, alignment, color, and spatial relationships collectively determine the harmony and balance of the smile. Among these teeth, the maxillary central incisors serve as the primary reference for esthetic evaluation, while the lateral incisors and canines contribute to the continuity and symmetry of the anterior dental arch. Even subtle alterations in the proportional relationship between these teeth may influence the overall perception of smile attractiveness, highlighting the importance of objective esthetic assessment during restorative and prosthodontic treatment planning [2,3].

Several mathematical concepts have been proposed to guide the evaluation and restoration of anterior tooth proportions. Of the various esthetic ratios, the Golden Proportion and the Recurring Esthetic Dental (RED) proportion have received the greatest attention in research [4]. Levin introduced the Golden Proportion based on the mathematical ratio of approximately 1.618:1, suggesting that, when viewed from the frontal aspect, the apparent width of the maxillary lateral incisor should be in golden proportion to that of the central incisor, and the apparent width of the canine should similarly relate to the lateral incisor [5]. Although this concept has been extensively applied in esthetic dentistry, subsequent investigations have questioned its universal applicability because natural dentitions frequently deviate from these idealized proportions [6]. In response to these limitations, Ward proposed the RED Proportion, which advocates a uniform percentage decrease in the visible width of successive anterior teeth as the dentition extends distally from the midline [7]. Unlike the Golden Proportion, the RED concept allows flexibility and accommodates individual variation, making it potentially more applicable to natural dentitions. Studies have reported RED values ranging from 62% to 80%, although considerable variability has been observed among different populations [8,9]. Evidence from various geographic and ethnic groups suggests that neither the Golden Proportion nor the RED Proportion can be considered universally applicable. Several studies have demonstrated a low prevalence of the Golden Proportion in natural dentition, whereas the occurrence of RED Proportion also varies substantially across populations [2,4,10]. A study conducted in Dakshina Kannada reported that only 27.8% of individuals demonstrated Golden Proportion, while RED Proportion was observed more frequently [2]. Similar observations have been reported in populations from Kerala, Saudi Arabia, Malaysia, and Iraq, indicating that anterior tooth proportions are influenced by ethnic and demographic characteristics rather than a single universal mathematical standard [4,11]. These findings support the need for population-specific reference values to facilitate predictable and individualized esthetic treatment planning.

Although several studies have evaluated anterior dental proportions in different populations, information regarding the Tirupati urban population remains unavailable. Considering the ethnic diversity of the Indian population, regional differences in dental morphology may influence the prevalence of these esthetic proportions. Establishing normative data for this population could therefore provide valuable guidance for prosthodontic rehabilitation, restorative procedures, and digital smile design. Furthermore, advances in digital photography and cast-based measurements now permit accurate and reproducible assessment of anterior tooth dimensions using non-invasive techniques [12]. Therefore, the present study aimed to evaluate the Golden Proportion and the RED proportion in an urban population of Tirupati using two distinct measurement methods and to assess their applicability for esthetic smile analysis in this population.

## Materials and methods

A one‑year cross‑sectional study (September 2024-September 2025) was conducted in the Department of Prosthodontics, CKS Theja Institute of Dental Sciences and Research, Tirupati, Andhra Pradesh. Ethical approval was obtained from the Institutional Ethics Committee (Approval No. CKS/PROSTHO/23‑24/01), and the study followed the Declaration of Helsinki guidelines. Written informed consent was collected from all participants, with confidentiality maintained and the option to withdraw at any time without affecting treatment.

The sample size was calculated using the single population proportion formula, considering an expected prevalence of 27.8% for the Golden Proportion based on a previous study [2], a 95% confidence level (Z = 1.96), and an absolute precision of 6.2%. The minimum calculated sample size was 200 participants, all of whom were included in the study.

A total of 200 individuals aged 18-30 years were recruited by convenience sampling from eligible patients and students attending the Department of Prosthodontics, CKS Theja Institute of Dental Sciences and Research, Tirupati. Eligible participants had fully erupted, natural maxillary anterior teeth within the esthetic zone. Facial symmetry was assessed clinically by frontal examination in the natural head position, and individuals with obvious facial asymmetry or craniofacial deformities were excluded. Additional exclusion criteria included a history of orthodontic treatment, anterior crowding, missing anterior teeth, maxillary midline diastema, peg-shaped lateral incisors, microdontia or other developmental anomalies affecting the maxillary anterior teeth, and the presence of restorations, crowns, veneers, or any prosthetic rehabilitation involving the anterior teeth. The prevalence of the Golden Proportion and RED Proportion was evaluated using two independent measurement techniques: standardized digital photographic analysis and maxillary dental cast analysis. Measurements obtained by both methods were subsequently compared.

Photographic analysis

Standardized frontal smile photographs were obtained under controlled indoor conditions using a Canon EOS 1500D DSLR camera (Canon Inc., Tokyo, Japan) mounted on a tripod. A fixed camera-to-subject distance of 30 cm was maintained for all participants, and frontal smile photographs were captured at 1:2 lens magnification without cheek retractors. Uniform illumination was achieved using two studio soft-box lights positioned bilaterally at approximately 45° to the participant to minimize shadows and surface reflections. A matte green background was used to eliminate background interference and standardize image contrast. Participants were photographed in a natural head position while maintaining a relaxed, natural smile. Full-face and frontal smile photographs were captured following the American Academy of Cosmetic Dentistry (AACD) photographic guidelines [13].

The images were saved in JPEG format at a resolution of 300 dpi and imported into Adobe Photoshop CC for analysis. The mesiodistal dimensions of the maxillary central and lateral incisors, along with the canines, were determined by marking the most incisal proximal contact points and measuring the distance between the selected reference landmarks. Tooth widths were initially recorded in pixels and converted to millimeters using an individualized calibration factor derived from the clinically measured mesiodistal width of the right maxillary central incisor. This calibration was performed solely to establish the pixel-to-millimeter conversion factor for each participant and to standardize image measurements. The calibrated right central incisor measurement was therefore not considered an independent validation of agreement between photographic and cast-based measurements. The pixel-to-millimeter conversion was performed using the following equation:

Millimeters = (Pixels ÷ DPI) × 25.4

where 25.4 represents the number of millimeters in one inch. The measured variables included the visible widths of the right and left maxillary central incisors (right central incisor width (CIWR) and left central incisor width (CIWL)), lateral incisors (right lateral incisor width (LIWR) and left lateral incisor width (LIWL)), and canines (right canine width (CAWR) and left canine width (CAWL)). To assess intra-examiner reliability, all image measurements were repeated by the same examiner after a two-week interval.

Irreversible hydrocolloid impressions of the maxilla were made and poured with Type IV dental stone to obtain casts. Positioned on a horizontal plane, the casts were used to measure the greatest mesiodistal widths of the anterior teeth with a digital Vernier caliper (precision 0.01 mm). Measurements for both right and left sides were recorded and entered into Microsoft Excel for subsequent statistical analysis.

The Golden Proportion was determined by comparing the visible width ratio of the lateral incisor to the central incisor and the canine to the lateral incisor with the theoretical value of 0.618. Ratios approximating this value were considered consistent with the Golden Proportion.

The RED Proportion was calculated using the following equations:

Lateral incisor width ÷ Central incisor width × 100

Canine width ÷ Lateral incisor width × 100

The RED proportion was determined by calculating the ratio of the lateral incisor to the central incisor and the canine to the lateral incisor for both sides. The calculated ratios were compared between the photographic and cast-based measurement methods and analyzed descriptively to evaluate the consistency of the proportional relationship. All image measurements were repeated by the same examiner after a two-week interval to minimize measurement variability and maintain consistency in the measurement procedure.

Statistical analysis

Data were analyzed using IBM SPSS Statistics for Windows, Version 27 (Released 2019; IBM Corp., Armonk, New York, United States). Continuous variables were reported as mean ± SD and categorical variables as frequencies and percentages. Paired t-tests compared photographic and caliper methods, as well as right and left anterior teeth. The Golden Proportion was assessed using lateral incisor-to-central incisor (LI/CI) and canine-to-lateral incisor (CA/LI) ratios, while the RED proportion was calculated as successive percentage ratios. Statistical significance was set at p < 0.05.

## Results

The study included 200 participants aged 18-30 years, comprising 116 male participants (58%) and 84 female participants (42%). The mean mesiodistal widths of the maxillary central incisors measured using the photographic and cast-based measurement methods showed no statistically significant differences (p > 0.05). However, as the right maxillary central incisor served as the calibration reference for photographic measurements, the observed agreement for this tooth was expected by design and should not be interpreted as independent evidence of method agreement. In contrast, the cast-based measurement method recorded significantly greater mesiodistal widths for the maxillary lateral incisors and canines than the photographic method (p < 0.001) (Table 1).

**Table 1:** Comparison of Mean Mesiodistal Widths of Maxillary Anterior Teeth Measured Using the Photographic and Cast-Based Measurement Method *Paired t-test; p < 0.05 indicates statistical significance. CIWL, left central incisor width; CIWR, right central incisor width; LIWL, left lateral incisor width; LIWR, right lateral incisor width; CAWL, left canine width; CAWR, right canine width.

Tooth	Photographic Method Mean ± SD (mm)	Cast-Based Measurement Method Mean ± SD (mm)	p-value
CIWL	10.22 ± 0.78	10.21 ± 0.78	0.842
CIWR	10.06 ± 0.83	10.05 ± 0.83	0.867
LIWL	6.31 ± 0.82	7.57 ± 1.02	<0.001*
LIWR	6.33 ± 0.57	7.60 ± 0.74	<0.001*
CAWL	4.92 ± 0.71	5.97 ± 0.87	<0.001*
CAWR	5.23 ± 0.61	6.34 ± 0.78	<0.001*

No statistically significant differences were observed between the right and left maxillary central incisors or lateral incisors (p > 0.05). However, the canine width differed significantly between the two sides (p = 0.018), although the absolute difference was small (Table 2).

**Table 2:** Comparison of Right and Left Mesiodistal Widths of Maxillary Anterior Teeth Measured Using the Photographic Method *Paired t-test; p < 0.05 indicates statistical significance.

Tooth	Left Mean ± SD (mm)	Right Mean ± SD (mm)	p-value
Central incisor	10.22 ± 0.78	10.06 ± 0.83	0.214
Lateral incisor	6.31 ± 0.82	6.33 ± 0.57	0.742
Canine	4.92 ± 0.71	5.23 ± 0.61	0.018*

Measurements obtained using the cast-based measurement method demonstrated no statistically significant differences between the right and left central incisors or lateral incisors (p > 0.05). A statistically significant difference was observed between the right and left canine widths (p = 0.012), although the magnitude of this difference was clinically small (Table 3).

**Table 3:** Comparison of Right and Left Mesiodistal Widths of Maxillary Anterior Teeth Measured Using the Vernier Caliper Method *Paired t-test; p < 0.05 indicates statistical significance.

Tooth	Left Mean ± SD (mm)	Right Mean ± SD (mm)	p-value
Central incisor	10.21 ± 0.78	10.05 ± 0.83	0.231
Lateral incisor	7.57 ± 1.02	7.60 ± 0.74	0.688
Canine	5.97 ± 0.87	6.34 ± 0.78	0.012*

The mean LI/CI ratio differed significantly between the photographic and cast-based measurement methods (p < 0.001). In contrast, no statistically significant difference was observed for the CA/LI ratio (p = 0.412) (Table 4).

**Table 4:** Comparison of Golden Proportion Ratios Obtained Using the Photographic and Vernier Caliper Methods *Paired t-test; p < 0.05 indicates statistical significance. LI/CI: lateral incisor-to-central incisor; CA/LI: canine-to-lateral incisor

Ratio	Photographic Method Mean ± SD	cast-based measurement method Mean ± SD	p-value
LI/CI	0.627 ± 0.09	0.753 ± 0.11	<0.001*
CA/LI	0.812 ± 0.10	0.820 ± 0.12	0.412

The mean RED proportion for the LI/CI ratio differed significantly between the photographic and cast-based measurement methods (p < 0.001). However, no statistically significant difference was observed between the methods for the CA/LI ratio (p = 0.458) (Table 5).

**Table 5:** Comparison of RED Proportion Obtained Using the Photographic and Vernier Caliper Methods *Paired t-test; p < 0.05 indicates statistical significance. LI/CI: lateral incisor-to-central incisor; CA/LI: canine-to-lateral incisor

Ratio	Photographic Method Mean ± SD (%)	Cast-Based Measurement Method Mean ± SD (%)	p-value
LI/CI	62.7 ± 9.0	75.3 ± 11.0	<0.001*
CA/LI	81.2 ± 10.0	82.0 ± 12.0	0.458

## Discussion

The maxillary anterior teeth are key determinants of smile esthetics, making their proportional relationships an important consideration in prosthodontic rehabilitation and esthetic dentistry. Although the Golden Proportion and RED Proportion have been proposed as mathematical guidelines for smile design, their applicability to natural dentition remains controversial because tooth dimensions vary among individuals and populations. The present study evaluated these proportions in the Tirupati urban population using photographic and Vernier caliper measurements to determine their clinical relevance.

The findings demonstrated that the maxillary anterior teeth did not consistently conform to either the Golden Proportion or RED Proportion, supporting previous studies that questioned the universal applicability of these mathematical concepts. These observations reinforce the influence of ethnic, anatomical, and population-specific variations on anterior tooth dimensions, suggesting that esthetic rehabilitation should be individualized rather than based solely on fixed numerical ratios [2,4,5].

A major finding of the present study was the significant difference between photographic and cast-based measurements for the lateral incisors and canines, whereas no statistically significant differences were observed for the central incisors. However, the agreement observed for the right maxillary central incisor should be interpreted with caution because it served as the calibration reference for converting photographic measurements from pixels to millimeters. Consequently, comparisons between the two methods are more appropriately interpreted based on the lateral incisors and canines, which were independent of the calibration procedure. The greater dimensions obtained using the Vernier caliper represent the actual anatomical mesiodistal widths, whereas photographic measurements reflect the apparent tooth widths perceived in the frontal view. This discrepancy is primarily attributed to dental arch curvature, tooth inclination, and perspective, which reduce the visible width of teeth located farther from the midline [8].

These findings are consistent with those of Gillen et al., Fayyad et al., and Agrawal et al., who reported that photographic measurements primarily reflect visual perception and are influenced by projection errors and the inherent limitations of two-dimensional imaging, whereas direct measurements provide more accurate anatomical dimensions [14-16]. In contrast, Hasanreisoglu et al. demonstrated that standardized photographic protocols can closely approximate direct measurements [17]. Nevertheless, even under standardized conditions, photographs cannot completely reproduce the three-dimensional morphology of the dental arch. Therefore, photographic analysis should be considered a valuable adjunct for esthetic evaluation rather than a replacement for direct anatomical measurements.

From a clinical perspective, these findings indicate that anatomical accuracy and visual perception are not always identical. While cast measurements are essential for restoration fabrication and diagnosis, photographic analysis better represents how a smile is perceived during social interaction. Consequently, combining both methods provides a more comprehensive approach to esthetic treatment planning.

The present study demonstrated no significant right-left differences in the mesiodistal widths of the central and lateral incisors, indicating good bilateral symmetry. In contrast, the canines showed a statistically significant but clinically small asymmetry. Similar findings have been reported by Alqahtani et al., who attributed canine variations to developmental factors, eruption patterns, functional adaptation, and tooth inclination [9]. These observations suggest that minor canine asymmetry represents normal anatomical variation rather than an esthetic discrepancy requiring correction, supporting the concept that overall harmony is more important than perfect symmetry.

Photographic analysis produced a lateral incisor-to-central incisor ratio closer to the theoretical Golden Proportion than cast-based measurements. Because this comparison was independent of the calibration reference tooth, it more accurately reflects differences between the two measurement methods. These findings demonstrate differences between photographic and cast-based measurements, with the photographic method yielding ratios numerically closer to the theoretical Golden Proportion.

The results agree with those of Al-Marzok et al. and Mahshid et al., who concluded that the Golden Proportion is rarely observed in natural dentition and should not be regarded as a universal esthetic standard [4,18]. Likewise, Londono et al. reported considerable variation in anterior tooth proportions among different populations, highlighting the influence of ethnic and anatomical diversity [19].

The RED proportion analysis demonstrated significant differences between the photographic and cast-based measurement methods for the lateral incisor-to-central incisor ratio, whereas no significant difference was observed for the canine-to-lateral incisor ratio. The variability in RED values indicates that a constant recurring proportion was not consistently present in the study population. These findings agree with those of Baghiana et al. and Azimi et al., who reported that RED proportion is not consistently observed in natural dentition and therefore lacks universal applicability [6,7]. Ward originally proposed the RED proportion as a flexible alternative to the Golden Proportion, emphasizing consistent proportional reduction rather than adherence to a fixed ratio [20]. Similarly, Rosenstiel et al. demonstrated that both clinicians and patients generally prefer harmonious tooth proportions over exact mathematical relationships [21]. Collectively, these findings suggest that RED proportion is best regarded as a flexible clinical guideline rather than a definitive esthetic standard.

Neither the Golden Proportion nor the RED proportion was consistently observed in the Tirupati urban population, highlighting the influence of ethnic and anatomical variation on anterior tooth dimensions. Similar findings have been reported in populations from the United Kingdom, Kenya, India, and the Middle East, where considerable variation in anterior tooth proportions limited the universal applicability of mathematical esthetic concepts [3,22-24]. Jouhar et al. further emphasized that esthetic perception is influenced by cultural and social preferences in addition to anatomical characteristics [25]. Although Lucchi et al. and Zarbah et al. suggested that modified proportional concepts may be useful in selected clinical situations, these approaches should be considered adjunctive rather than universal principles [26,27].

The findings of the present study have important implications for prosthodontic and esthetic practice. Photographic analysis reflects the apparent tooth dimensions perceived during smiling, whereas cast-based measurements provide accurate anatomical dimensions required for diagnosis and restoration fabrication. As the right maxillary central incisor was used for photographic calibration, method comparison is most appropriately interpreted using the lateral incisors and canines. Therefore, integrating both methods provides a more comprehensive approach to esthetic treatment planning.

The limited applicability of the Golden Proportion and RED proportion further emphasizes that mathematical ratios should serve as reference guidelines rather than rigid treatment objectives. Instead, clinicians should base smile design on individual patient characteristics, including facial morphology, tooth anatomy, ethnic background, and esthetic expectations.

Future studies involving larger and more diverse populations are required to establish population-specific normative data for anterior tooth proportions. The application of three-dimensional digital technologies, including intraoral scanners and cone-beam computed tomography, may improve measurement accuracy by overcoming the limitations of two-dimensional photographic analysis. Longitudinal studies evaluating age-related changes and investigations correlating tooth proportions with facial morphology and patient-reported esthetic outcomes would further strengthen evidence-based smile design. The present study has certain limitations. It was conducted at a single center and included only young adults from the Tirupati urban population with well-aligned natural dentition. Participants with crowding, spacing, previous orthodontic treatment, or anterior restorations were excluded to standardize measurements, which may limit the generalizability of the findings. Photographic calibration was based on the mesiodistal width of the right maxillary central incisor; therefore, agreement for this tooth was expected by design and should not be interpreted as an independent comparison between the two measurement methods. In addition, photographic analysis was limited to two-dimensional images, and all measurements were performed by a single examiner without blinding, which may have introduced observer bias. Formal intra-examiner reliability statistics were not calculated. Furthermore, only mesiodistal tooth widths were evaluated, while other esthetic parameters and observer-based esthetic assessments were not included. Finally, the cross-sectional design precludes evaluation of changes in tooth proportions over time.

## Conclusions

Within the limitations of this study, neither the Golden Proportion nor the RED Proportion was consistently observed in the maxillary anterior dentition of the Tirupati study population. Cast-based measurements recorded significantly greater mesiodistal widths for the lateral incisors and canines than photographic measurements, whereas no statistically significant difference was observed for the central incisors. These findings indicate that photographic and cast-based measurement methods provide complementary information and should be interpreted according to their respective applications. The results further support an individualized approach to esthetic treatment planning, in which mathematical proportions are used as reference guidelines rather than rigid standards. Additional studies involving larger and more diverse populations and three-dimensional digital measurement techniques are recommended to establish population-specific normative data and improve the clinical applicability of anterior tooth proportion analyses.
